# Visible to near-infrared refractive properties of freshly-excised human-liver tissues: marking hepatic malignancies

**DOI:** 10.1038/srep27910

**Published:** 2016-06-14

**Authors:** Panagiotis Giannios, Konstantinos G. Toutouzas, Maria Matiatou, Konstantinos Stasinos, Manousos M. Konstadoulakis, George C. Zografos, Konstantinos Moutzouris

**Affiliations:** 1 Laboratory of Electronic Devices and Materials, Department of Electronic Engineering, Technological Educational Institution of Athens, Athens, Greece; 2 First Department of Propaedeutic Surgery, Hippocration Hospital, Athens Medical School, Athens, Greece

## Abstract

The refractive index is an optical constant that plays a significant role in the description of light-matter interactions. When it comes to biological media, refraction is understudied despite recent advances in the field of bio-optics. In the present article, we report on the measurement of the refractive properties of freshly excised healthy and cancerous human liver samples, by use of a prism-coupling technique covering the visible and near-infrared spectral range. Novel data on the wavelength-dependent complex refractive index of human liver tissues are presented. The magnitude of the real and imaginary part of the refractive index is correlated with hepatic pathology. Notably, the real index contrast is pointed out as a marker of discrimination between normal liver tissue and hepatic metastases. In view of the current progress in optical biosensor technologies, our findings may be exploited for the development of novel surgical and endoscopic tools.

Determining the refractive index of biological matter is needed for biophotonics applications including laser therapy, optical diagnosis and biomedical imaging. Recently, a limited number of studies have also pointed towards the direct use of refractive index as a marker for distinguishing between normal and malignant human breast and prostate^1^, as well as rat brain tissues^2^. Despite its obvious significance, reports on the refractive index of tissues remain scarce in literature. This fact may be attributed to complications associated with the available experimental techniques, as well as to the notion of refractive index itself which, in the case of tissues, is not straightforward.

For homogeneous transparent media, the refractive index is a readily determined real number that quantifies the apparent reduction of the speed of light inside the medium, relative to the speed of light in vacuum. Tissues, however, are both absorbing and highly inhomogeneous, comprising internal arbitrarily-shaped structures in the macroscopic (≥mm) and microscopic (say, 50 nm to 50 μm) scale, the latter giving rise to photon multiple scattering effects^3^,^4^,^5^. As a consequence, tissue refractive index is a spatially fluctuating complex number, the imaginary part of which incorporates absorption and scattering.

To analyze the micro-optical properties of tissues, the concept of the “effective” refractive index has been introduced within the frame of an effective medium theory^6^. This approach is based on the assumption that a tissue may be treated as an optically turbid medium, whose refractive index equals the sum of its mean (“effective index”) and a relatively small spatially varying part. Consequently, a laser beam propagating through tissue carries a coherent component, which corresponds to the “average” electromagnetic field and decreases with penetration depth, as well as a diffuse component, which corresponds to the local fluctuations of the electromagnetic field around its average. Under the preceding assumptions, the tissue effective refractive index may be thought of as the microscopically-averaged refractive index experienced by the coherent light component. In that sense, the macro-optical properties of tissues may be adequately described by use of the effective index, which in turn exhibits spatial variations reflecting inhomogeneities in the macroscopic scale.

Efforts to experimentally determine the refractive properties of biological samples ranging from cell cultures to human tissues have surfaced at a high rate in recent years. On the grounds of their operating principle, the available techniques fall into two basic categories, namely imaging and critical angle approaches. Imaging methods, based on interferometry and/or microscopy, have been used for the determination of the real part of tissue effective indices^2^,^7^,^8^,^9^,^10^,^11^,^12^, as well as for mapping the microscopic fluctuations of the real index in one^13^, two^1^,^14^ and three^15^ dimensions. Critical angle methods are unsuited for resolving the microscopic index variations, but they accommodate the determination of the effective refractive indices^16^,^17^,^18^,^19^,^20^,^21^,^22^ with several advantages: First, they have been proven suitable for the determination of both real and imaginary part of the effective index^17^,^22^. Second, they may be coupled to multiple laser sources without major experimental reconfiguration, so that the spectral dependence of the refractive index may be readily evaluated^17^,^22^. Furthermore, they require low-cost instrumentation, are experimentally straightforward in terms of calibration and alignment and may be used with thick, untreated, non-fixed tissues, thus exhibiting great potential for rapid-evaluation applications in medical practice.

In the present study, a fully-automated prism coupling refractometer operating in the reflection geometry and equipped with five laser sources is utilized for the determination of the complex effective indices of freshly excised, non-fixed, human liver tissues. Measurements are presented for five different wavelengths in the visible and near-infrared, enabling the evaluation of spectral dispersion effects. A total number of 30 liver tissue samples were investigated within 2 hours after surgical removal from 17 patients, including cancer free individuals, as well as patients diagnosed with hepatocellular carcinoma (HCC) or liver metastases from other gastrointestinal primary sites. The data accumulated herein provide strong evidence that the refractive index contrast is a high-potential marker of hepatic malignancies. Based on this evidence, currently growing optical biosensor technologies^23^,^24^,^25^ may lead to the development of novel endoscopic and surgical evaluation tools.

## Materials and Methods

### Sampling and tissue preparation

Tissue specimens from the liver of 17 patients who underwent surgical treatment were investigated in this study. All individuals provided their informed consent, while all methods were carried out in accordance with the guidelines approved by the administrative board (IRB) of the Hippocration General Hospital of Athens. Four samples were surgically excised from four patients who were not associated to cancer or any other disease that could adversely affect the liver parenchyma (reference group, N). Ten samples of non-cancerous liver tissue (NMET) and ten metastatic samples (MET) were also excised from ten patients with liver metastases. Finally, three samples of non-cancerous liver tissue (NHCC) and three HCC samples were excised from three patients diagnosed with hepatocellular carcinoma, yielding a total of 30 specimens (Table 1).

**Figure 1:**
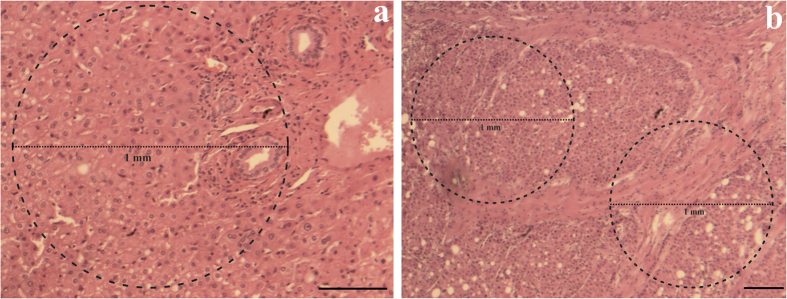
H&E staining of two tissue samples included in the study; magnified 100X (**a**) and 40X (**b**).Magnification bar = 250 μm. Normal liver parenchyma with portal space on the right (**a**) and a section taken from a liver with G2 HCC (**b**). Circle dotted lines are indicative of the area on each sample covered by the laser beam (1 mm diameter).

Immediately after surgical excision, the samples were stored on ice in Ringer’s solution and slices with a typical surface of 5 mm × 5 mm and a thickness of approximately 2 mm were manually prepared under a dissecting stereoscope. The *ex-vivo* measurements (including tissue preparation) lasted approximately 10 minutes and were concluded within a timeframe of not more than 2 hours after surgical removal of each specimen. Subsequent histological examination with standard haematoxylin and eosin (H&E) staining revealed that the cell morphology of the samples remained intact after the experimental procedure (Fig. 1). The histopathological reports for adjacent tissue biopsies were used to further assess and confirm the pathological status of each specimen examined.

### Experimental setup

**Figure 2:**
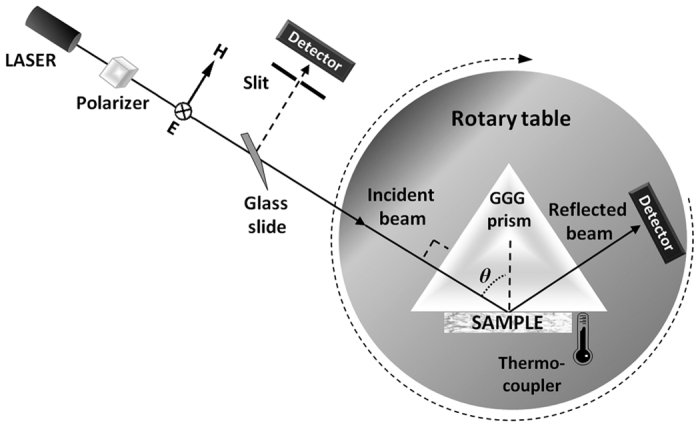
The experimental setup. Top-view of the prism-coupling refractometer, inhere depicted at normal incidence with respect to the prism’s entrance facet. Five independent laser sources are used sequentially. A polarizer ensures the s-polarization of the input beam (that is, perpendicular electric (**E**) and parallel magnetic (**H**) field vector, with respect to the plane of incidence). A photodiode detects the reflected light from the prism/tissue interface. The prism and the detector stand on a computer-driven rotary table. A second detector, along with a slit and a glass slide, is used for angle referencing.

The experimental setup is an extended version of the Metricon 2010/M model^26^, Metricon Corp., Pennington, N.J., USA (Fig. 2). The sample under investigation is attached to the base of a gadolinium gallium garnet (GGG) reference prism of known refractive index *n*_*p*_, forming spontaneously a high-quality interface, without applying external pressure or including an exogenous intermediate liquid. Radiation from a continuous-wave laser is directed upon the base of the prism at an angle of incidence that can be controlled via a computer-driven rotary table, with an angle resolution of 0.025°. A photodetector measures the angular variation of the reflected beam intensity from the prism/tissue interface. Each scan for the acquisition of a single reflectance profile is concluded within 75 seconds. The apparatus utilizes five independent laser sources, including three diode lasers emitting radiation at 450 nm, 964 nm and 1,551 nm, as well as a helium–neon laser and a frequency-doubled YAG laser emitting radiation at 632.8 nm and 532 nm, respectively. A polarizer is employed to ensure the s-polarization of the input laser beams, which are delivered unfocused, with a diameter in the order of 1 mm. When switching from one laser source to the next, a calibration routine is followed so as to determine the reference angle. This self-referencing procedure monitors the parasitic reflection from the prism entrance facet, by combined use of an additional photodiode and a pinhole. A thermocoupler is employed for measuring the temperature at the prism base, which is expected to equal the temperature of the sample.

### Data analysis

**Figure 3:**
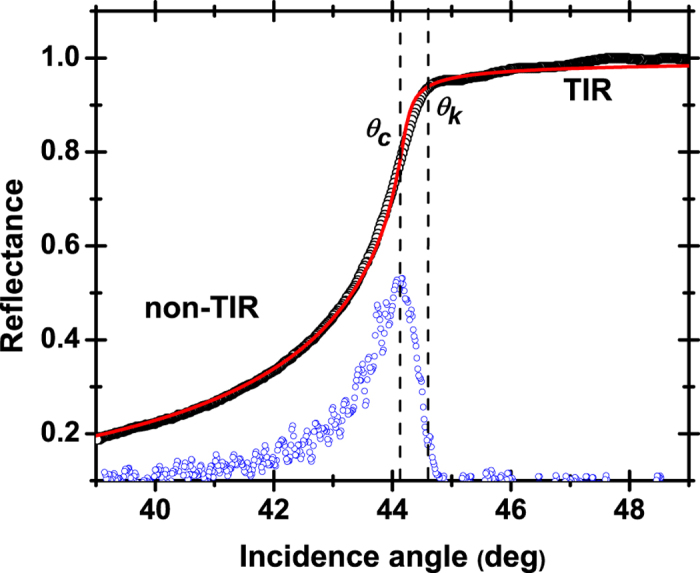
An indicative reflectance profile and the corresponding data analysis. Raw reflectance data (black circles) along with the first derivative of reflectance (blue circles, arbitrary units) and Fresnel fit (red line). The position of the knee, as well as the critical angle, is denoted. Data correspond to 964 nm.

A reflectance profile *R*(*θ*) = *I*_*r*_/*I*_*i*_ (*I*_*r*_ and *I*_*i*_ are the reflected and incident beam intensities, respectively) collected by use of the present setup at 964 nm for a tissue specimen is shown in (Fig. 3). The profile exhibits two distinctive regions^27^, one towards the largest angles, corresponding to the attenuated total internal reflection (TIR) regime and one towards the smallest angles (non-TIR or NTIR), where part of the incident beam refracts into tissue. The transition from one region to the other is relatively gradual, a feature that is typical of turbidity^25^.

To analyze the shape of the collected profiles, three different approaches were cross-evaluated. In a first instance, the pattern recognition algorithm of Metricon Corp., which is a standard accessory of the 2010/M model, identifies the characteristic angle of the “knee” in the reflectance curve. The knee angle *θ*_*k*_ is located inside the TIR region-in the vicinity of the transition point-and signals a sudden increase in the attenuation of the TIR reflectance as the incidence angle decreases, a behavior that can be traced back to the Goos–Hänchen effect. In a second step, the differentiation total internal reflection (DTIR) method^19^,^28^,^29^ was employed. DTIR is based on the determination of the critical angle *θ*_*c*_ corresponding to the peak in the first derivative of the reflectance profile. In reasonable proximity to *θ*_*k*_, the critical angle *θ*_*c*_ marks the transition from the TIR to the NTIR regime, so that by use of Snell’s law:


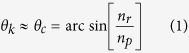


In the last equation, *n*_*r*_ is the real part of the complex refractive index *n*_*s*_ of the sample, which also comprises the imaginary component *n*_*i*_ :  *n*_*r *_= *n*_*s*_ + *i · n*_*i*_. In a third and final step, raw data were analyzed by use of Fresnel theory^17^,^22^,^27^,^30^, which is known to sufficiently describe reflectance of optically clear, as well as turbid media. Accounting for s-polarized light, the Fresnel equation reads:


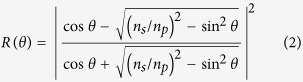


With transparent and homogeneous materials (*n*_*i*_ = 0), reflectance is a discontinuous function of incidence angle and the transition from the-non-attenuated in this case-TIR to the NTIR region is sharp. As a consequence, *θ*_*k*_ coincides with *θ*_*c*_ and equation (1) deduces directly from equation (2). In other words, the three methods described previously may be used equally well to produce the same value of the sample’s index (*n*_*s*_ = *n*_*r*_), with an accuracy reaching fourth decimal digit by use of the present experimental setup. However, in the presence of turbidity (*n*_*i*_ ≠ 0), the situation is not as straightforward. To begin with, as is seen in (Fig. 3), the apparent angles *θ*_*k*_ and *θ*_*c*_ are now at a distance, losing their concrete interpretation and turning their determination into a heuristic process. Moreover, standard Fresnel theory combined with common fitting tools, leads to progressively inaccurate computation of the refractive constants, as turbidity increases.

In an effort to short out the turbidity induced ambiguities, estimated values of *n*_*r*_ were accumulated by use of all three alternative approaches. DTIR process was based on derivative calculation from discrete data by averaging the slopes of the two lines connecting each data point with its two adjacent points. Fresnel fit was performed with the differential evolution algorithm as implemented in Wolfram Mathematica 10 with default options and the results were post-processed by a quasi-Newton algorithm (BFGS). Data fit was robust in the sense that other algorithms (e.g Nelder-Mead and simulated annealing) and option values other than the default did not improve the results.

Along this line, a total number of 450 reflectance profiles where examined (30 tissue specimens × 5 wavelengths × typically 3 measurements at different sample locations). The pattern recognition algorithm failed to identify the angle of the knee in about half of the cases. In general, discrepancies in the estimation of *n*_*r*_ between the Fresnel fit and DTIR were in the order of 3 × 10^−3^, seemingly higher in the visible and smaller in the infrared. In comparison, the pattern recognition algorithm overestimated *n*_*r*_ by as much as 10^−2^. Despite the differences, all three methods were able to distinguish between different pathological conditions with more or less comparable efficiency.

As a result of the preceding observations, we opted the use of the Fresnel analysis which can be assumed to generate in a non-subjective manner estimates on both *n*_*r*_ (with a safely presumed accuracy reaching third decimal digit) and *n*_*i*_. This assumption is reinforced by the fact that the measured reflectance profiles were fitted to the Fresnel equation with R^2^ > 0.09 in nearly all 450 cases.

## Experimental Results

### Complex refractive index of the various tissue groups

For each one of the available specimens and a given wavelength, typically three reflectance profiles were collected, each one corresponding to different locations on the surface of the sample, so as to account for macroscopic tissue inhomogeneity; the number of experimental runs was restricted to three, so as to minimize measurement time and thus, tissue dehydration or other subsequent tissue damaging effects. During the experimental procedure, temperature was monitored but not actively stabilized therefore data correspond to room conditions (24 °C ± 2 °C). Via Fresnel analysis, a complex refractive index was calculated for each experimental run. Taking into account the mm-diameter of the laser beams, this result is directly interpreted as the microscopically-averaged *effective* index, thereafter referred to simply as “refractive index”.

Subsequently, mean values of the wavelength-dependent complex refractive index were computed for the five different tissue groups, namely N, MET, NMET, HCC and NHCC, by averaging over all measurements corresponding to same pathology specimens. The results of this inter-individual analysis, along with the respective standard deviation (SD) values, are given in (Table 2). The mean real indices were fitted to the standard three-term Cauchy equation (3), while corresponding imaginary indices were fitted to the power law of equation (4):


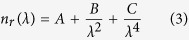



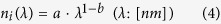


**Figure 4:**
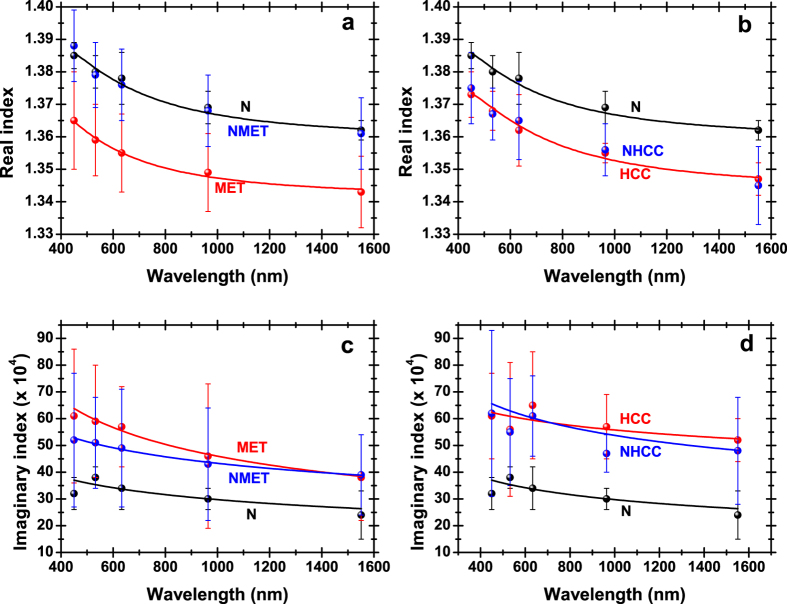
Wavelength-dependent refractive index for the various tissue groups. Real (**a,b**) and imaginary (**c,d**) indices as a function of wavelength. Dots correspond to inter-individually averaged experimental data. Lines depict theoretical fits. Designated colors represent the different tissue groups. Error bars indicate SD.

The fitting coefficients (*A*, *B*, *C*, *a* and *b*) are also shown in (Table 2), while averaged index data and corresponding theoretical curves are plotted in (Fig. 4). Three sets of Cauchy coefficients are sufficient for modeling the wavelength dependent real index of the five tissue groups (N & NMET, MET, NHCC & HCC) with an *R*^2^ > 0.98. Similar fit quality for the imaginary indices is only achieved for the largely populated MET and NMET groups. For the N, HCC and NHCC tissue collections, equation (4) provides only a rough approximation of the wavelength-scaling trends.

Several interesting conclusions may be deduced from the information accumulated in (Table 2) and (Fig. 4). Malignant tissues (MET and HCC) exhibit significantly lower real, and higher imaginary, indices compared to the reference group (N). Liver tissues at the normal state excised from metastatic patients (NMET) exhibit real indices that are practically identical with the reference group (N) and significantly different from the metastasis itself (MET). This picture reverses in the case of primary liver cancer: the real indices of the NHCC group depart from N and are practically indistinguishable from HCC. A similar comparison for the imaginary indices reveals that both NMET and NHCC groups are quite close to MET and HCC, respectively, exhibiting significant differences from the N group. In summary, the following self-explanatory scheme applies:


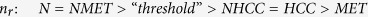



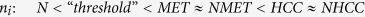


where the “threshold”, separating the normal from the diseased state, could correspond to a real index of 1.36 and an imaginary index of 0.0037 at an indicative wavelength of 964 nm.

The preceding observations may be compiled in order to draw the following “map”: In the absence of cancer, the refractive properties of the liver are “uniform”, with the real index resting above (and the imaginary below) some “threshold” value. In the presence of metastasis, the organ exhibits “nearly uniform” and above “threshold” values of *n*_*i*_, while the real index fluctuates above and below “threshold”, marking the passage from normal regions to lesions. In the presence of a primary tumor, the organ exhibits nearly “uniform” refractive properties, with *n*_*r*_ resting below and *n*_*i*_ above “threshold”, making the distinction between malignant and non-malignant regions impractical. In that sense, the most direct implication of the present study revolves around the use of the real index for marking the metastases.

This statement is further supported by one way ANOVA analysis [F(2,103) = 36.35] (performed using GraphPad Prism version 5.00 for Windows, GraphPad Software, San Diego California USA) showing that there is a significant effect of the real index change across the N, NMET and MET groups included in our study at the p < 0.05 level (this results refer to measurements at 964 nm, while identical effects were observed at all wavelength range). Post hoc comparisons using the Tukey’s test indicated that the NMET vs MET and N vs MET groups varied significantly at the p < 0.05 level, while the N vs NMET comparison was not significant. Before discussing in more detail the discrimination of the metastatic state, it is noted that the complex refractive index data and the corresponding fits may be used to assess several additional tissue optic effects. Such an exemplary study is attempted below.

### Light extinction estimates

The wavelength dependent imaginary index relates to the extinction coefficient *μ* (that is, the sum of the absorption coefficient *μ*_*a*_ and the scattering coefficient*μ*_*s*_) according to:


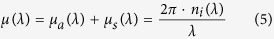


Tissue scattering follows a power low as a function of wavelength and prevails^31^,^32^ over tissue absorption, which manifests its presence through localized spectral features^3^. It is thus possible that the signature of absorption is imprinted in (Fig. 4), in the cases of N, HCC and NHCC groups, for which the straightforward power-scaling law of equation (4) is unable to provide highly accurate fits. Taking into account the dominant role of scattering, equations (4) and (5) lead to the following approximation:


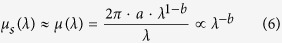


The so-called “scattering power” *b* in equation (6) has been previously studied for liver specimens^33^,^34^, while an averaged scattering power for numerous soft tissues (including liver) has also been calculated^32^. In the preceding references, reported values of *b* were 1.05, 1.64 and 1.286 ± 0.521, respectively, in good agreement with *b* estimates produced in the present work (Table 2).

### Marking the metastasis

**Figure 5:**
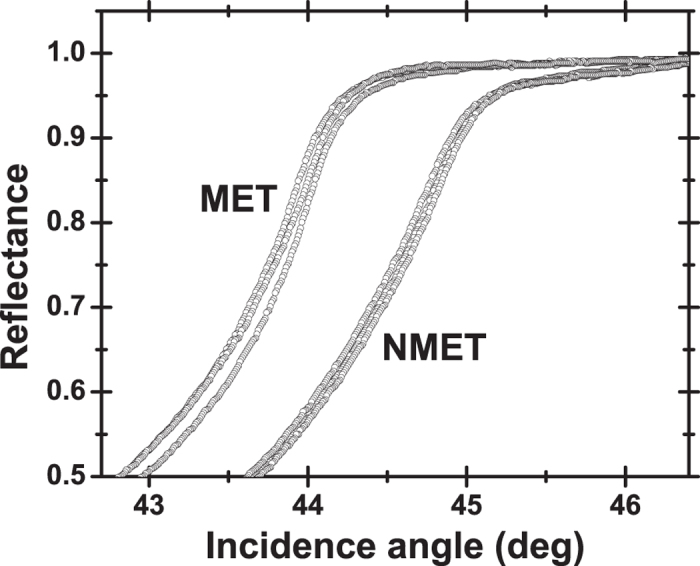
Reflectance profiles from a single patient’s MET and NMET tissues. Raw data of reflectance as a function of incidence angle corresponding to three different locations of a MET and an NMET tissue from the same patient. In this exemplary case, the 1,551 nm laser source is used.

Despite the strong trends that have been already demonstrated, it is still doubtful that a “threshold” index can be defined as a standalone indicator of liver metastasis, particularly when accounting for the variance of the refractive indices, quantified by error bars in Fig. (3). It is therefore meaningful to cross-compare the reflectance profiles of sample pairs (MET and NMET) excised from the same metastatic patient (Fig. 5). This comparison reveals a pathology-induced horizontal shift in reflectance, observed systematically for all five wavelengths of measurement, all three experimental runs and all 10 metastatic patients.

**Figure 6:**
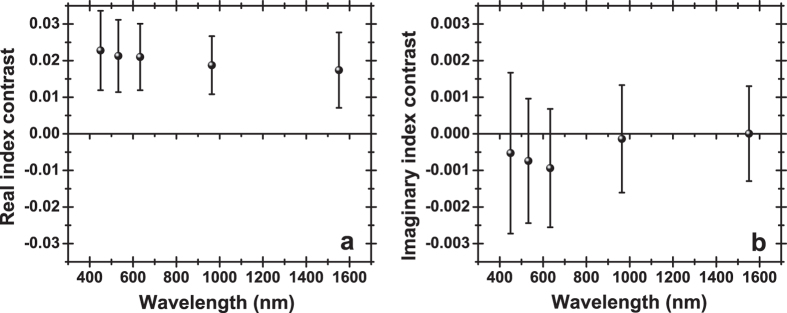
Averaged real (**a**) and imaginary (**b**) index contrast between normal tissue and lesions on a diseased liver. Averaging takes place over the 10 metastatic patients. Error bars indicate SD.

To gain a further insight, MET and NMET “intra-tissue” mean indices (real and imaginary) were calculated by averaging over the (typically) three measurements at a given wavelength. Consequently, a real and an imaginary index contrast was calculated for each one of the ten metastatic patients, defined as the difference between the real and imaginary “intra-tissue” mean indices of the respective MET and NMET specimens. Averaged values over the ten patients of the real and imaginary index contrast, along with the respective SD, are shown in (Fig. 6).

Not surprisingly, the systematic shift in the reflectance curves is clearly mirrored in the real, rather than the imaginary, index contrast. This effect is attributed to the fact that the real index regulates the horizontal position of the reflectance profile, while the imaginary index dictates the shape of the TIR to NTIR transition zone. As a consequence, the real index contrast seen on a particular diseased organ emerges as a promising method to set apart normal liver regions from metastatic tissues.

On the contrary, the imaginary index contrast exhibits only a trend of slightly negative values in the visible spectral range, approaching zero towards the near infrared. At any given wavelength, patients with opposite signs of imaginary index contrast are found, thus this parameter is not a conclusive indicator of tissue pathology. It is noted that in a previous study^31^ based on homogenized, rather than fresh non-treated tissue samples, the extinction coefficient of normal liver was found to be about twice that of the metastases, suggesting that a similar relationship should also apply for the imaginary indices. Comparing this observation with our own investigation indicates that the homogenization process alters significantly the scattering properties of tissues.

### Index variance considerations

Trends on the inter-individual variance of the refractive index may be recognized, accounting for (a) its real and imaginary part (b) the effect of wavelength (c) the effect of the pathological condition. To access these trends, the coefficient of variation CV is calculated as the ratio of SD over the mean by use of data in Table (2). To begin with, CV is found to be as small as <1% for the real index, increasing by one order of magnitude (or more) for the imaginary. This observation supports the integrity of the real index as a marker of disease and may be attributed either to a preferential efficiency of our method to resolve the location-rather than the shape-of the transition in reflectance, or more likely to the inherent optical properties of tissues. A modest wavelength dependence of CV is observed, with slightly smaller variance noticed typically in the infrared, compared to visible wavelengths. On the contrary, a non-negligible change of CV is seen for different pathologies. More specific, the real index inter-individual variance increases from a wavelength averaged value of 0.36% for the reference group (N), to 0.47% for the primary tumor patients (HCC) and 0.77% for the metastatic patients (MET). To ensure that this behavior is not caused simply by the different sizes of the respective samples, we also calculated the relative standard error for the various tissue groups, which was found to scale equivalently (N < HCC < MET). The real index inter-individual variance within the N group follows the intrinsic differences in the hepatic tissue organization, which is supposedly lower compared to the malignant states. The inclusion of patients with underlying liver pathologies, namely HBV infection, Non Alcoholic Fatty Liver Disease and cirrhosis, also contributes to the increased variance of the malignant (MET and HCC) groups with respect to N. Several additional factors may contribute to the fact that the highest variance is seen within the metastatic histological type. On one hand, the cell populations forming the metastatic tissue originate from different types of primary adenocarcinomas of the gastrointestinal tract (in the metastatic group of patients, 4 individuals with primary tumor sites at the transverse colon, 3 at the sigmoid colon, 1 at the descending colon, 1 at the ascending colon and 1 individual with metastatic cancer of the pancreas were included in this study). On the other hand, 7 out of 10 metastatic patients had received preoperative chemotherapy. Selective pressure induced by therapeutic agents to subpopulation of metastatic cells could also result in increased intra-and inter-tumoral heterogeneity^35^,^36^.

As regards to the intra-tissue real index variance, some useful trends may also be seen. Indicatively, the CV of the (typically) three measurements performed at a given wavelength on a specific MET or NMET tissue sample is found to be smaller than the respective inter-individual CV by a factor of approximately four. This factor remained practically unchanged, when we increased the number of experimental runs from three to ten, by use of the largest available MET and NMET tissue specimens (with an 8 × 8 mm surface). These observations reinforce further the view that real index variations from one person to another are largely attributed to inter-individual tissue heterogeneity, while within the same individual, significant real index differences (in the order of 0.02) may only indicate drastic structural changes, like those occurring in the presence of metastasis.

## Discussion

During the past decades, the groundwork was laid for a much needed database containing the optical constants of human tissues. Nevertheless, this effort remained unsystematic and inconsistent, with just a handful of articles reporting relevant data in the bibliography^1^,^7^,^8^,^15^,^16^,^21^,^22^ under variant experimental specifications (Table 3). In the present work, we provide for the first time to the best of our knowledge data on the complex refractive index of non-fixed non-dehydrated human tissues from an internal organ, at multiple laser wavelengths covering the visible and near infrared. Under similar conditions, there exists only one previous report for human skin^22^, in which case however, due to the substantial extinction of light in this tissue type, external pressure was applied to the specimen. Furthermore, our investigation included a sample size that is significantly larger than previous works, enabling a useful estimation of the refractive index variability. The reported data (and corresponding fits) can be used to evaluate secondary tissue optic parameters, optimize theoretical models of light transport in tissue and solve problems concerning interfaces between an optical head and a biological specimen, thus adding specificity to numerous imaging modalities^37^ and improving phototherapy strategies^38^,^39^. Most notably, however, our investigation revealed the correlation of the complex refractive index with liver pathology, clearly indicating that the real index contrast is a promising marker of metastasis.

Moreover, the present study revealed two main phenomenologies, the first one relating to the higher real (and lower imaginary) indices of cancerous tissues, with respect to the liver’s normal state. In an effort to interpret the real index lowering effect of cancer, it has to be acknowledged that in a cellular scale, this optical constant is typically assumed to be higher for cancer cells than for normal cells^40^. In a macroscopic scale, however, the tumor consists of more than a mass of cells^41^ and its structural properties do not resemble healthy tissue’s architecture. In that sense, the tumor mass shows altered collagen levels^42^,^43^,^44^, cell count and increased water content^45^, the latter known to result in real index reduction^36^. In the particular case of the metastatic disease, where the most significant lowering effect is observed, the differential histological pattern of the metastatic tissue compared to the normal hepatic parenchyma should also be considered. On the other side, the cancer-induced imaginary index increase should directly reflect a corresponding increase in scattering. The latter trend has been previously observed for breast malignancies^46^, while exactly the opposite has been reported in the case of skin cancer^47^. It thus appears that the imaginary index exhibits inherent sensitive dependences on multiple parameters, making the complete interpretation of its behavior an elusive task that is beyond the scope of the present work.

The second phenomenology accounts for the observation that a diseased organ may exhibit either uniform (or nearly uniform) refractive properties, as is the case with HCC, or significantly non-uniform properties, as is the case with the real index of metastatic individuals. While non-uniformity may be straightforwardly explained in terms of the drastic tissue alterations due to the disease, the cases where uniformity is observed could indicate that the corresponding optical constant depends strongly on other parameters too. Not coincidentally, uniformity is observed particularly in the case of HCC that is often associated with underlying inflammatory chronic liver diseases^48^,^49^,^50^,^51^, provoking tissue damage and fluid attenuation that could change the optical properties of the affected organ as a whole.

From a medical perspective, neoplastic pathologies of the liver represent a primary issue in clinical oncology nowadays^52^,^53^. The liver is a common site of metastases, particularly from colorectal carcinoma, while it is estimated that up to two-thirds of patients with colorectal liver metastases ultimately die from their disease^54^. When applied as a treatment of choice for hepatic malignancies, surgical resection has been shown to result in improved survival outcomes^55^,^56^. Even in the case of the hepatic tissue, that is known for its regenerative potential, minimizing surgical trauma and enabling the intra-operative assessment of the resection margins^57^, remain important determinants of survival.

As a result, the surgeon needs direct feedback during a procedure with respect to the pathological characteristics of the resected and the remaining tissue, prior to the standard of the pathology report, which usually takes several days to obtain. Tissue refractive index and its apparent differences in normal and malignant states seem to be a very promising marker that could be exploited in this direction. In that sense, existing biosensor technologies capable of detecting refractive index changes with high accuracy, could lead to a reliable simple to use and cost effective margin assessment tool.

## Additional Information

**How to cite this article**: Giannios, P. *et al*. Visible to near-infrared refractive properties of freshly-excised human-liver tissues: marking hepatic malignancies. *Sci. Rep*. **6**, 27910; doi: 10.1038/srep27910 (2016).

## Figures and Tables

**Table 1:** Summary of tissue selection.

Patient condition	Number of individuals	Tissue pathology	Number of tissue specimens
Non-cancerous	4	Normal liver tissue (*N*)	4
Liver metastases	10	Metastatic tissue (*MET*)	10
		Non-cancerous tissue (*NMET*)	10
Hepatocellular carcinoma	3	Primary liver tumor (*HCC*)	3
		Non-cancerous tissue (*NHCC*)	3

**Table 2:** Mean refractive indices of the various tissue groups and corresponding fit parameters.

Tissue	Mean real index ± SD				
	450 nm	532 nm	632.8 nm	964 nm	1551 nm
N	1.385 ± 0.004	1.380 ± 0.005	1.378 ± 0.008	1.369 ± 0.005	1.362 ± 0.003
MET	1.365 ± 0.015	1.359 ± 0.011	1.355 ± 0.012	1.349 ± 0.012	1.343 ± 0.011
NMET	1.388 ± 0.011	1.379 ± 0.010	1.376 ± 0.011	1.368 ± 0.011	1.361 ± 0.010
HCC	1.373 ± 0.007	1.368 ± 0.006	1.362 ± 0.011	1.355 ± 0.003	1.347 ± 0.005
NHCC	1.375 ± 0.011	1.367 ± 0.008	1.365 ± 0.012	1.356 ± 0.008	1.345 ± 0.012
**Tissue**	**10**^**4**^** × [Mean imaginary index ± SD]**				
	**450 nm**	**532 nm**	**632.8 nm**	**964 nm**	**1551 nm**
N	32 ± 6	38 ± 4	34 ± 8	30 ± 4	24 ± 9
MET	61 ± 25	59 ± 21	57 ± 15	46 ± 27	38 ± 16
NMET	52 ± 25	51 ± 17	49 ± 22	43 ± 21	39 ± 15
HCC	61 ± 16	56 ± 25	65 ± 20	57 ± 12	52 ± 8
NHCC	62 ± 31	55 ± 20	61 ± 15	47 ± 7	48 ± 20
**Tissue**	**Real index fit parameters**			**Imaginary index fit parameters**	
	**A**	**B (μm**^**2**^)	**C (μm**^**4**^)	**a**	**b**
N	1.35910	0.00827	−0.000576	196	1.27
MET	1.34127	0.00634	−0.000324	780	1.41
NMET		Same as N		238	1.25
HCC	1.34348	0.00998	−0.000793	145	1.14
NHCC		Same as HCC		301	1.25

**Table 3:** Review of articles containing data on the refractive index of human tissues, along with corresponding experimental details.

Human Tissue			Number of samples	Wavelength (nm)	Ref.
Type	Status	Pathology			
Liver	Freshly excised	Normal & Malignant	30	450, 532, 632.8, 964, 1,551	This work
Liver	Treated^(a)^	–	6^(d)^	633	[21]
Liver	Treated^(b)^	–	≤2	632.8	[16]
Skin	*In vivo*	–	1	980	[15]
Skin	*In vivo*	–	1	1,300	[7]
Skin	*In vivo*	–	2	1,300	[8]
Skin	Freshly excised^(c)^	–	12	325, 442, 532, 633, 850, 1,064, 1,310, 1,557	[22]
Skin	Treated^(a)^	–	1	1,300	[7]
Skin	Treated^(a)^	–	4^(d)^	633	[21]
Kidney	Treated^(a)^	–	6^(d)^	633	[21]
Kidney	Treated^(b)^	–	≤2	632.8	[16]
Breast	Treated^(b)^	Normal & Microcalcified	2	White light	[1]
Prostate	Treated^(b)^	Normal & Malignant	18	White light	[1]
Mesenteric adipose	Treated^(a)^	–	1	1300	[7]
Cardiac muscle	Treated^(a)^	–	1	1300	[7]
Myocardium	Treated^(a)^	–	6^(d)^	633	[21]

^(a)^Dehydrated/postmortem, ^(b)^fixed, ^(c)^under pressure ≥10^5^ Pa, ^(d)^three of which from fetus. This collection excludes former studies on the refractive index of human fluids (e.g., blood plasma and aqueous humor).
